# The Remarkable Metrological History of Radiocarbon Dating [II]

**DOI:** 10.6028/jres.109.013

**Published:** 2004-04-01

**Authors:** Lloyd A. Currie

**Affiliations:** National Institute of Standards and Technology, Gaithersburg, MD 20899-8370 U.S.A.

**Keywords:** accelerator mass spectrometry, apportionment of fossil and biomass carbon, “bomb” ^14^C as a global tracer, dual isotopic authentication, metrological history, molecular dating, radiocarbon dating, the Turin Shroud, SRM 1649a

## Abstract

This article traces the metrological history of radiocarbon, from the initial breakthrough devised by Libby, to minor (evolutionary) and major (revolutionary) advances that have brought ^14^C measurement from a crude, bulk [8 g carbon] dating tool, to a refined probe for dating tiny amounts of precious artifacts, and for “molecular dating” at the 10 µg to 100 µg level. The metrological advances led to opportunities and surprises, such as the non-monotonic dendrochronological calibration curve and the “bomb effect,” that gave rise to new multidisciplinary areas of application, ranging from archaeology and anthropology to cosmic ray physics to oceanography to apportionment of anthropogenic pollutants to the reconstruction of environmental history.

Beyond the specific topic of natural ^14^C, it is hoped that this account may serve as a metaphor for young scientists, illustrating that just when a scientific discipline may appear to be approaching maturity, unanticipated metrological advances in their own chosen fields, and unanticipated anthropogenic or natural chemical events in the environment, can spawn new areas of research having exciting theoretical and practical implications.

## 1. Introduction

This article is about metrology, the science of measurement. More specifically, it examines the metrological revolutions, or at least evolutionary milestones that have marked the history of radiocarbon dating, since its inception some 50 years ago, to the present. The series of largely or even totally unanticipated developments in the metrology of natural ^14^C is detailed in the several sections of this article, together with examples of the consequent emergence of new and fundamental applications in a broad range of disciplines in the physical, social, and biological sciences.

The possibility of radiocarbon dating would not have existed, had not ^14^C had the “wrong” half-life—a fact that delayed its discovery [1]. Following the discovery of this 5730 year (half-life) radionuclide in laboratory experiments by Ruben and Kamen, it became clear to W. F. Libby that ^14^C should exist in nature, and that it could serve as a quantitative means for dating artifacts and events marking the history of civilization. The search for natural radiocarbon was itself a metrological challenge, for the level in the living biosphere [ca. 230 Bq/kg] lay far beyond the then current state of the measurement art. The following section of this article reviews the underlying concepts and ingenious experimental approaches devised by Libby and his students that led to the establishment and validation of the “absolute” radiocarbon technique.

That was but the beginning, however. Subsequent metrological and scientific advances have included: a major improvement in ^14^C decay counting precision leading to the discovery of natural ^14^C variations; the global tracer experiment following the “pulse” of excess ^14^C from atmospheric nuclear testing; the growing importance of quantifying sources of biomass and fossil carbonaceous contaminants in the environment; the revolutionary change from decay counting to atom counting (AMS: accelerator mass spectrometry) plus its famous application to artifact dating; and the demand for and possibility of ^14^C speciation (molecular dating) of carbonaceous substances in reference materials, historical artifacts, and in the natural environment.

## 2. The Birth of Radiocarbon Dating

**Fig. 1:**
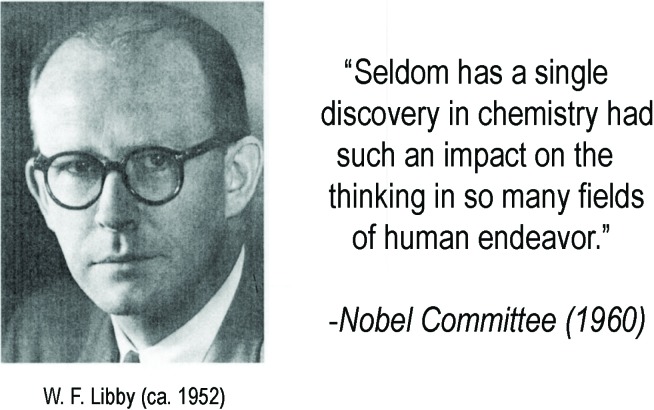
Portrait of W. F. Libby, about the time of publication of the first edition of his monograph, *Radiocarbon Dating* (1952), and statement of the Nobel Committee (1960) [3].

The year before last marked the 50th anniversary of the first edition of Willard F. Libby’s monograph, *Radiocarbon Dating*—published in 1952 [2]. Eight years later Libby was awarded the Nobel Prize in Chemistry. In a very special sense that small volume (111 pages of text) captured the essence of the path to discovery: from the initial stimulus, to both conceptual and quantitative scientific hypotheses, to experimental validation, and finally, to the demonstration of highly significant applications. The significance of Libby’s discovery, from the perspective of the Nobel Committee, is indicated in Fig. 1, which includes also a portrait of Libby in the year his monograph was published [3].^1^ The statement of the Nobel Committee represents an unusual degree of foresight, in light of unsuspected scientific and metrological revolutions that would take place in ensuing years.

**Fig. 2:**
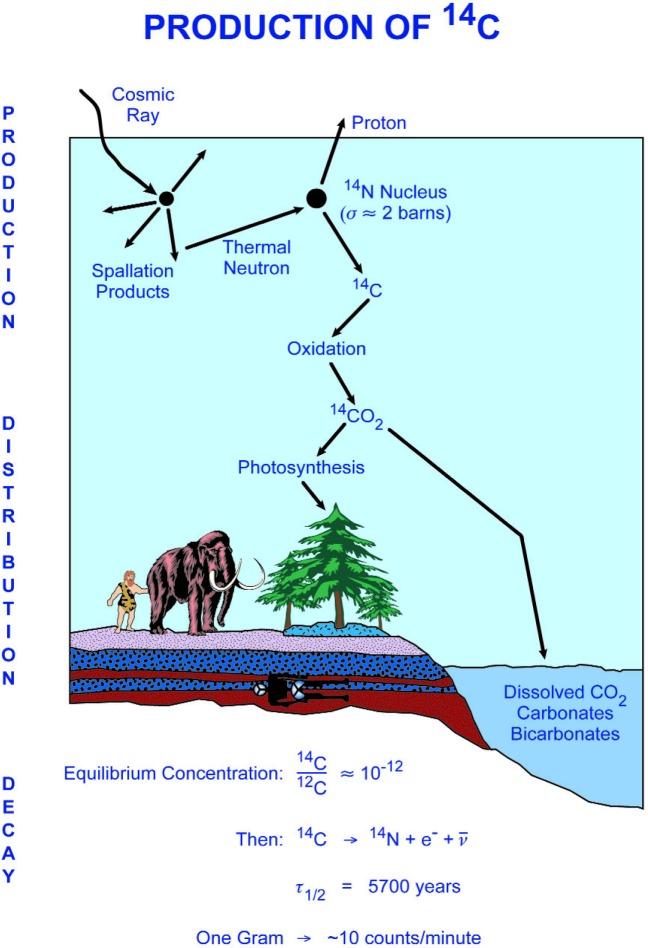
Graphical representation of the production, distribution, and decay of natural ^14^C (courtesy of D. J. Donahue).(Parameter values are approximate.)

Like many of the major advances in science, *Radiocarbon Dating* was born of Scientific Curiosity. As noted by Libby in his Nobel Lecture, “it had its origin in a study of the possible effects that cosmic rays might have on the earth and on the earth’s atmosphere” [4]. Through intensive study of the cosmic ray and nuclear physics literature, Libby made an important series of deductions, leading to a quantitative prediction of the natural ^14^C concentration in the living biosphere. As reviewed in chapter I of Libby’s monograph, and in the Nobel Lecture, the deductive steps included: (1) Serge Korff’s discovery that cosmic rays generate on average about 2 secondary neutrons per cm^2^ of the earth’s surface per second; (2) the inference that the large majority of the neutrons undergo thermalization and reaction with atmospheric nitrogen to form ^14^C via the nuclear reaction ^14^N(n,p)^14^C; (3) the proposition that the ^14^C atoms quickly oxidize to ^14^CO_2_, and that this mixes with the total exchangeable reservoir of carbon in a period short compared to the ca. 8000 year mean life of ^14^C. Based on the observed production rate of neutrons from cosmic rays (ca. 2 cm^−2^ s^−1^), their near quantitative transformation to ^14^C, and an estimate of the global carbon exchangeable reservoir (8.5 g/cm^2^), Libby estimated that the steady state radioactivity concentration of exchangeable ^14^C would be approximately [(2 × 60)/8.5] or about 14 disintegrations per minute (dpm) per gram carbon (ca. 230 mBq g^−1^). Once living matter is cut off from this steady state, exponential nuclear decay will dominate, and “absolute dating” will follow using the observed half-life of ^14^C (5568 years).^2^ Two critical assumptions are needed for absolute ^14^C dating: constancy of both the cosmic ray intensity and size of the exchangeable reservoir on average for many thousands of years. A graphical summary of the above points is given in Fig. 2.

Libby first postulated the existence of natural ^14^C in 1946, at a level of 0.2 to 2 Bq/mol carbon (1 dpm/g to 10 dpm/g) [5]. His first experimental task was to demonstrate this presence of “natural” ^14^C in living matter. The problem was that, even at 10 dpm/g, the ^14^C would be unmeasurable! The plan was to search for natural ^14^C in bio-methane, but the background of his well-shielded 1.9 L Geiger counter (342 counts per minute) exceeded the expected signal by a factor of 400. Libby and coworkers did succeed in demonstrating the presence of ^14^C in living matter, however. For an account of their creative approach to the problem, see their one page article in *Science*, “Radiocarbon from Cosmic Radiation” [6].^3^

**Fig. 3:**
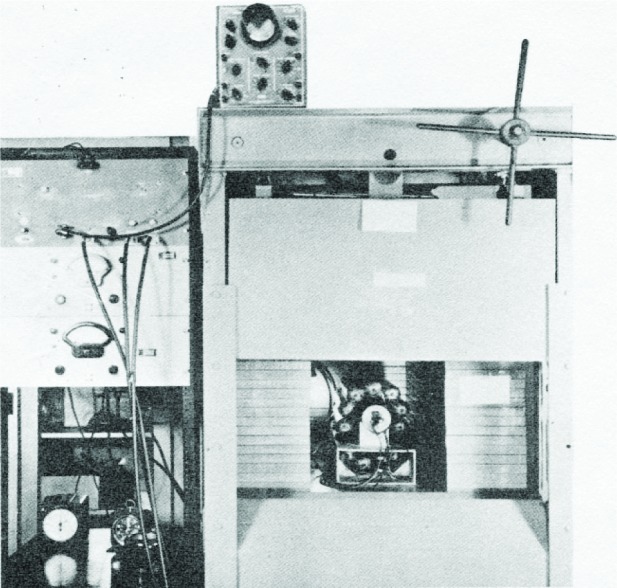
Low-level anticoincidence counting apparatus devised by Libby for the original ^14^C measurements that led to the establishment of the radiocarbon dating technique (Ref. [2], and *Radiocarbon Dating* (jacket cover) R. Berger and H. Suess, eds., Univ. California Press, Berkeley (1979).)

Having detected ^14^C in the living biosphere, Libby and his colleagues had to develop a measurement technique that was both quantitative and practical. The thermal diffusion enrichment technique [6] was not: it demanded very large samples and thousands of (1946) US dollars “to measure the age of a single mummy” [4]. Development of an acceptable technique was formidable, as outlined in Table 1. A substantial increase in signal was achieved by converting the sample to solid carbon, which coated the inner wall of a specially designed “screen wall counter;” but the background/signal ratio (16:1) still eliminated the possibility of meaningful measurements. At this point, Libby had an inspiration, from the analysis of the nature of the background radiation [4]. He concluded that it was primarily due to secondary, ionizing cosmic radiation having great penetrating power—negative mu mesons (µ^−^). By surrounding the sample counter with cosmic ray guard counters operating in an anti-coincidence mode, most of the µ^−^ counts could be eliminated, resulting in a further background reduction by a factor of twenty, to approximately 5 counts per minute (cpm). The final background to signal ratio of 0.8 for living carbon, made possible the measurement of natural (biospheric) ^14^C with a precision under 2 % (Poisson relative standard deviation) with a total (sample, background) counting time of just 2 d ([2], Chap. V). Fig. 3 shows the low-level counting apparatus devised by Libby, with which the seminal ^14^C dating measurements were made. The ^14^C screen wall counter is visible through the open, 8 inch thick cantilevered steel doors having a wedge-like closure. The steel “tomb” reduces the background by about a factor of five. The bundle of anticoincidence cosmic ray guard counters, seen surrounding the central counter in the figure, eliminates some 95 % of the residual background from the penetrating µ^−^ radiation, through electronic cancellation.

Perhaps the most valuable metrological lesson from Libby’s early work was the extreme importance of formulating a realistic theoretical estimate for the sought-after “signal.” Without that as a guideline for designing a measurement process with adequate detection or quantification capabilities, there is essentially no possibility that natural radiocarbon could have been found by chance with the then current radiation instrumentation.

### 2.1 Standards and Validation

Once the measurement of natural ^14^C became feasible, the immediate task tackled by Libby and his colleagues was to test the validity of the radiocarbon dating model. The first step consisted of determining the zero point of the natural radiocarbon decay curve— i.e., the radioactivity concentration (dpm ^14^C per gram C) in living matter, and to test for significant geographic variation. This was a major component of the PhD thesis of E. C. Anderson [7]; the result (*R*_o_) was (15.3 ± 0.5) dpm/g [255 Bq/kg] with no significant deviation from the hypothesis of a uniform global distribution.^4^ The next step was to measure the ^14^C concentrations in selected historical artifacts of known age, and compare them to the “absolute” ^14^C age. The latter was accomplished by comparing the artifact ^14^C concentration (dpm/g C) to that of the living biosphere. The absolute age derives from the inversion of first order nuclear decay relation, using 15.3 dpm/g and 5568 a as the parameters of the “absolute” natural ^14^C decay curve.

**Fig. 4:**
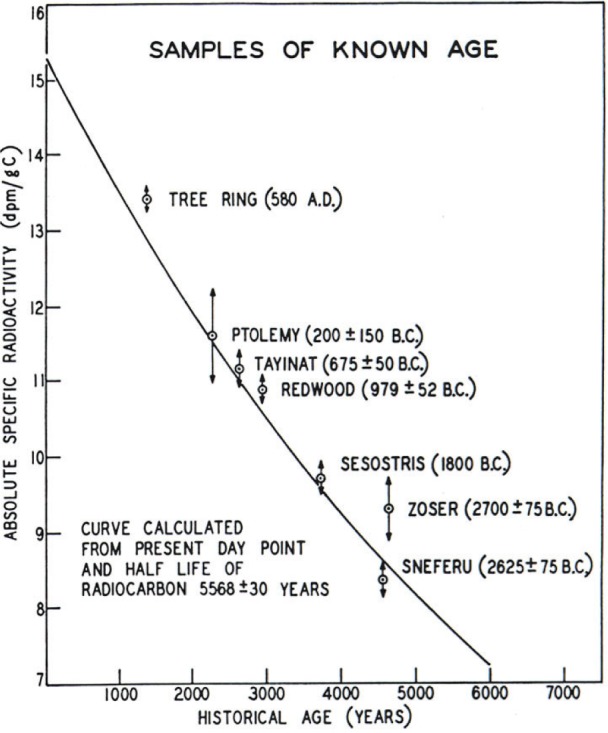
Radiocarbon dating validation curve (1952): the “curve of knowns” that first demonstrated that absolute radiocarbon dating “worked.” The validation points represent tree rings and historical artifacts of known age. The exponential function is *not fit to the data*, but derived from the independently measured half-life and the ^14^C content of living matter ([2], Fig. 1).

The famous result, utilizing known age tree rings and independently-dated Egyptian artifacts, is shown in Chapter I of Libby’s 1952 monograph and Fig. 4 in this article. Although the relative measurement uncertainties are moderately large (ca. 1 % to 5 %), the data provide a striking validation for the radiocarbon dating method over a period of nearly 5000 years. Note that the curve shown is *not fit to the data*! Rather, it represents the absolute, two-parameter nuclear decay function. (See [8] for detailed information on the validation samples selected.)

This initial absolute dating function served to establish the method, but it indicated the need for a universal radiocarbon dating standard, since the reference value for the intercept (here 15.3 dpm/g) would vary among laboratories, if they each made their own standards. The problem was tackled by the international radiocarbon community in the late 1950s, in cooperation with the U.S. National Bureau of Standards. A large quantity of contemporary oxalic acid di-hydrate was prepared as NBS Standard Reference Material (SRM) 4990B. Its ^14^C concentration was ca. 5 % above what was believed to be the natural level, so the standard for radiocarbon dating was defined as 0.95 times the ^14^C concentration of this material, adjusted to a ^13^C reference value of −19 per mil (PDB). This value is defined as “modern carbon” referenced to AD 1950. Radiocarbon measurements are compared to this modern carbon value, and expressed as “fraction of modern” (*f*_M_); and “radiocarbon ages” are calculated from *f*_M_ using the exponential decay relation and the “Libby half-life” 5568 a. The ages are expressed in years before present (BP) where “present” is defined as AD 1950. A published estimate for the ^14^C concentration of “modern carbon” is given as (13.53 ± 0.07) dpm/g [9]. In July 1983, a replacement SRM 4990C was substituted for the nearly exhausted SRM 4990B. It was prepared from oxalic acid derived from the fermentation of French beet molasses from harvests of 1977. A copy of the Certificate Analysis of SRM 4990C, together with pertinent references, may be obtained from the website: *http://nist.gov/srm* [10].^5^

Libby’s successful development of the science of radiocarbon dating led to the rapid establishment of more than a hundred dating laboratories world-wide, the initiation of a journal supplement that later became the journal Radiocarbon, and the establishment of a continuing series of triennial RADIOCARBON conferences, the first of which took place in Andover, Massachusetts in 1954.

## 3. Natural Variations

Already, by the time the Nobel Prize was awarded, Radiocarbon Dating appeared to be approaching maturity, with a rich future in application as opposed to new fundamental discovery. This all changed, however, when some of the fundamental assumptions proved to be invalid—what might be considered as the “failure of Radiocarbon Dating.”

This “failure” resulted from basic advances in ^14^C metrology. New approaches to low-level counting yielded measurement imprecision that ultimately approached 0.2 % (rsd);^6^ and construction of the “radiocarbon dating calibration curve” from meticulously counted annual tree ring segments showed that assumptions of constancy within different geochemical compartments of the exchangeable carbon reservoir, and over time, were invalid. (This is a classic example demonstrating that one cannot prove the “null hypothesis;” the validation curve that established the radiocarbon dating method demonstrated consistency (validity) only within the errors (uncertainties) of the validation measurements.) *The failure of the absolute dating model was, in fact, a notable success.* The revolutionary discovery of natural radiocarbon variations literally arose out of the “noise” of absolute radiocarbon dating, and it transformed the study of natural ^14^C into a multidisciplinary science, giving rise to totally new scientific disciplines of ^14^C solar and geophysics.

**Fig. 5:**
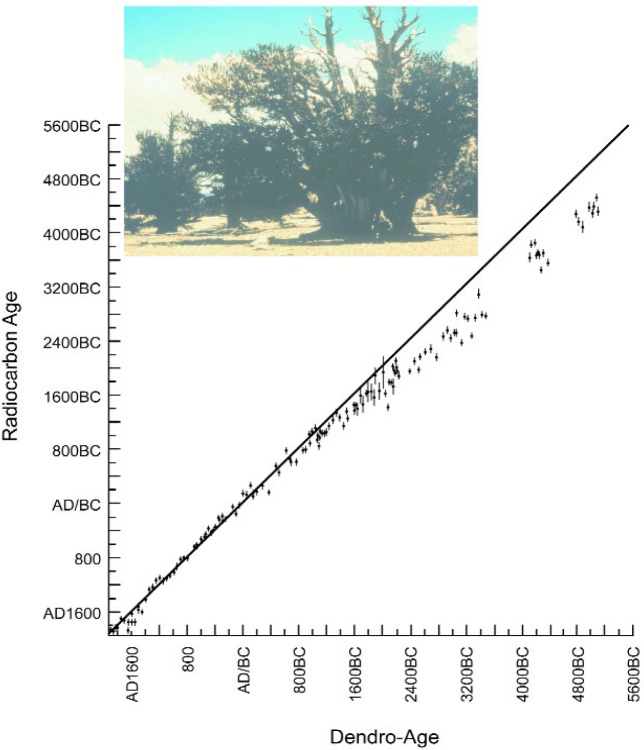
Radiocarbon Variations, discovered by comparison of high precision radiocarbon “dates” with high (annual) accuracy tree ring dates. The plot, which covers the period from about 5000 BC to the present, represents an early version of the radiocarbon dating calibration curve ([12], p. 110). The photo shows the Bristlecone pine, the major source of dendrodates extending back many millennia (Photo is courtesy of D. J. Donahue).

At his opening address at the 12th Nobel Symposium on *Radiocarbon Variations and Absolute Chronology* [12] in Uppsala, Nobelist Kai Siegbahn emphasized that “This subject is [now] interesting to specialists in many different fields, as can be seen from the list of participants, showing archaeologists, chemists, dendrochronologists, geophysicists, varved-clay geologists, and physicists” (Ref. [12], pp. 19f). An early version of the dendrochronological ^14^C calibration curve, presented by Michael and Ralph at the Symposium, is given in Fig. 5 (Ref. [12], p. 110).^7^ The Bristlecone pine, as shown in the figure, has made a seminal contribution to the science of dendrochronology, and through that, to the study of natural ^14^C variations. It is considered by some to be the world’s “oldest living thing,” with a single tree containing annual rings going back 4000 years or more. It is clear from Fig. 5 that the dendrochronological age shows a significant departure from the absolute ^14^C (nuclear) age, beginning about three thousand years ago, and continuing through the end of this series of measurements (ca. 5000 BC). These newly discovered deviations from the absolute dating model, of course, posed new scientific questions: what are the causes of the deviations, and can we use them to better understand Nature? In fact, the dendro-calibration curve serves dual purposes. For more classic “dating” disciplines, such as archaeology, anthropology, and geology (event dating), it gives an empirical correction function for the simple radiocarbon ages (BP) derived from the first order decay relation. For solar and geophysics and related disciplines, it gives the potential for the quantitative investigation of the causes of the variations.

The Nobel Symposium serves as a rich resource for information about the natural ^14^C variations. An excellent exposition of the three prime causative factors is given by Hans Suess (Ref. [12], pp. 595–605). These are: “(1) changes in the ^14^C production rate due to changes in the intensity of the [earth’s] geomagnetic field; (2) … modulation of the cosmic-ray flux by solar activity; (3) changes in the geochemical radiocarbon reservoirs and rates of carbon transfer between them.” The major departure (ca. 10 %) seen in Fig. 5 is considered to be due to the geomagnetic field, corresponding to a factor of two change in its intensity over the past 8000 years [15]. This has given major impetus to the science of archaeomagnetism. The other two factors are considered responsible for the partly periodic fine structure exhibited in the curve, with varying amplitudes of about 1 % to 2 %. (See Figs. 1, 2 in the Suess article, respectively, for plots of the first order (geomagnetic) and second order (fine structure) deviations from the ideal exponential decay function (“radiocarbon age”).)

**Fig. 6:**
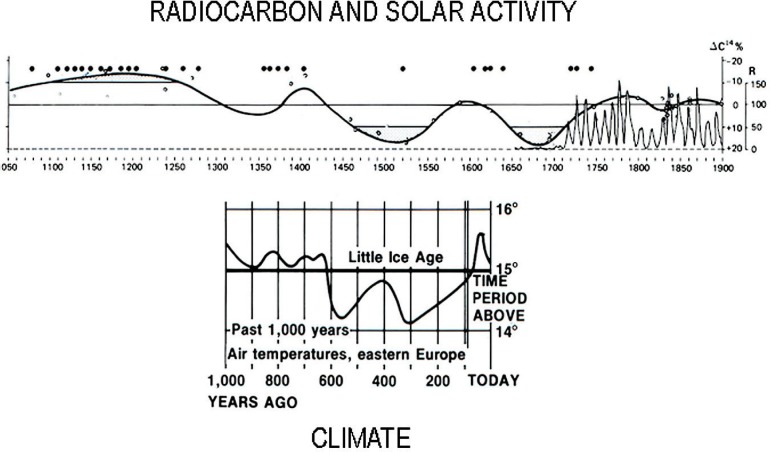
Radiocarbon Variations and Climate: the influence of solar activity (sunspot record) (top) on ^14^C concentrations (cosmic ray production rates) and climate (Maunder Minimum temperature record) (bottom) [15, 16].

A fascinating link exists between dendrochronology and radiocarbon age, related to climate. That is, tree rings by their width time series, like ice cores by their ^18^O time series, give insight into ancient climate [16]. This, in turn, may be linked to the aforementioned ^14^C variations from changing solar activity and/or variations in geochemical reservoirs. Fig. 6 represents a famous example of the inter-relationships among solar activity (sunspots), natural radiocarbon variations, and climate (Ref. [15], Fig. 5a; Ref. [16], p. 615). The upper part of the figure shows the correlation between the sunspot record (circles, and ca. 11 year cycles) and the ^14^C variations. The period of low solar activity, and correspondingly increased ^14^C activity, peaking at about 1500 AD and 1700 AD is striking. The lower part of the figure suggests a strong link to global climate, represented here by the “little ice age.”

## 4. The Bomb

Atmospheric nuclear testing had an unintended but profound impact on ^14^C geoscience. It approximately doubled the ^14^C concentration in atmospheric CO_2_, and consequently in living matter, by the mid-1960s. This came about because neutrons released from nuclear fission (or fusion) react with atmospheric nitrogen by exactly the same reaction, ^14^N(n,p)^14^C, as the secondary neutrons from cosmic rays. The “bomb pulse” of excess ^14^C was recorded in all parts of the living biosphere, from vintage wine [17] to contemporary tree rings [18]. It was characterized by a sharp injection of ^14^C in the early 1960s, followed by relatively slow geochemical decay after the limited (atmospheric) nuclear test ban treaty. Totally new and unanticipated opportunities to perform global tracer experiments resulted from this sudden, widespread injection of anthropogenic ^14^C into the biogeochemical system.

### 4.1 Excess ^14^C as a Global Geochemical Tracer

**Fig. 7:**
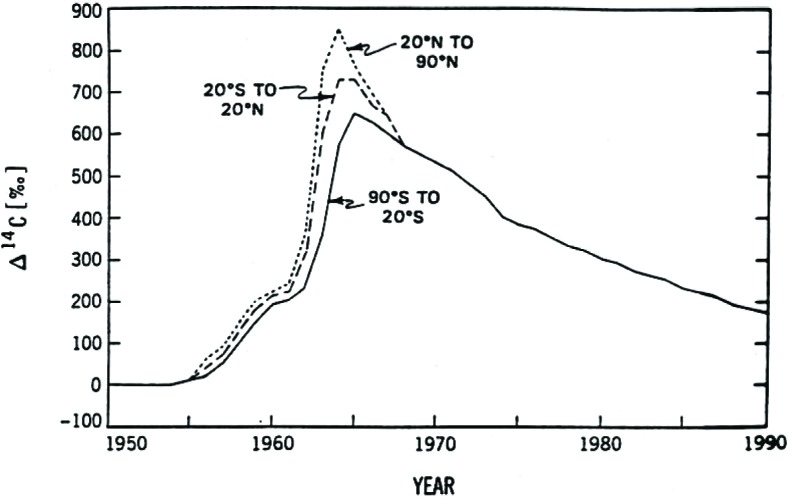
Input function of excess (“bomb”) ^14^C: a global tracer for carbon cycle dynamics in the atmosphere, biosphere, and oceans [19].

An extensive world-wide program of monitoring the excess atmospheric ^14^CO_2_ began with the onset of nuclear testing and continues today. Results of precise measurements of the input function for excess ^14^CO_2_ are shown in Fig. 7 (Ref.[19]; Ref. [20], Chap. 31, (I. Levin, et al.)). Use of this known pulse of excess ^14^C as a tracer has allowed scientists to study exchange and transport processes in the atmosphere, the biosphere, and the oceans on a scale that would otherwise have been nearly impossible. Simple visual examination of Fig. 7 shows, for example, that the excess atmospheric ^14^C injected in the northern hemisphere gave an attenuated signal in the southern hemisphere, and that there was a lag time of approximately 2 years.

**Fig. 8:**
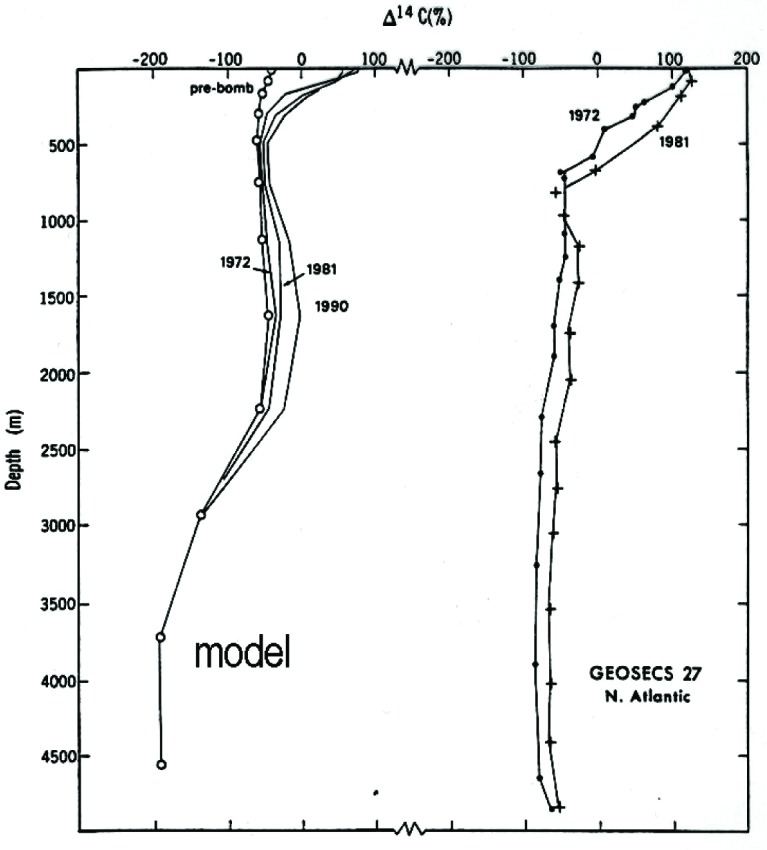
Excess ^14^C and ocean circulation (GEOSECS). Model (left) and experimental (right) vertical transects of bomb ^14^C in the North Atlantic [19].

Nowhere has the bomb pulse been more important than in furthering our understanding of the dynamics of the ocean. A comprehensive program (GEOSECS: Geochemical Ocean Section Study) to follow the plume of excess ^14^C as it diffused in the Atlantic and Pacific oceans was initiated in the 1970s. A small example of the findings is given in Fig. 8, where we find a nearly uniform distribution below the mixed layer, indicating rapid vertical transport in the North Atlantic, in contrast to model predictions [19, 21]. The scientific impact of this massive tracer study of ocean circulation is striking, considering, for example, the new knowledge it brings regarding the effects of the oceans on pollutant and heat transport and climate [22].^8^

### 4.2 The Second (Geochemical) Decay Curve of ^14^C: Isotopic-Temporal Authentication

Geochemical relaxation of the excess atmospheric ^14^C after about 1970 has resulted in a second (short-lived) “decay curve” for ^14^C (tail of the input function, Fig. 7). This has made possible a new kind of radiocarbon dating, where modern artifacts and forgeries, food products, forensic biology samples, and industrial bio-feedstocks can be dated with near annual resolution [24]. As a result of the new submilligram measurement capability (Sec. 6), short-term radiocarbon dating is beginning to achieve commercial importance, as exemplified by its application to the dual isotopic (^13^C, ^14^C) fingerprinting and time stamping of industrial materials.

**Fig. 9:**
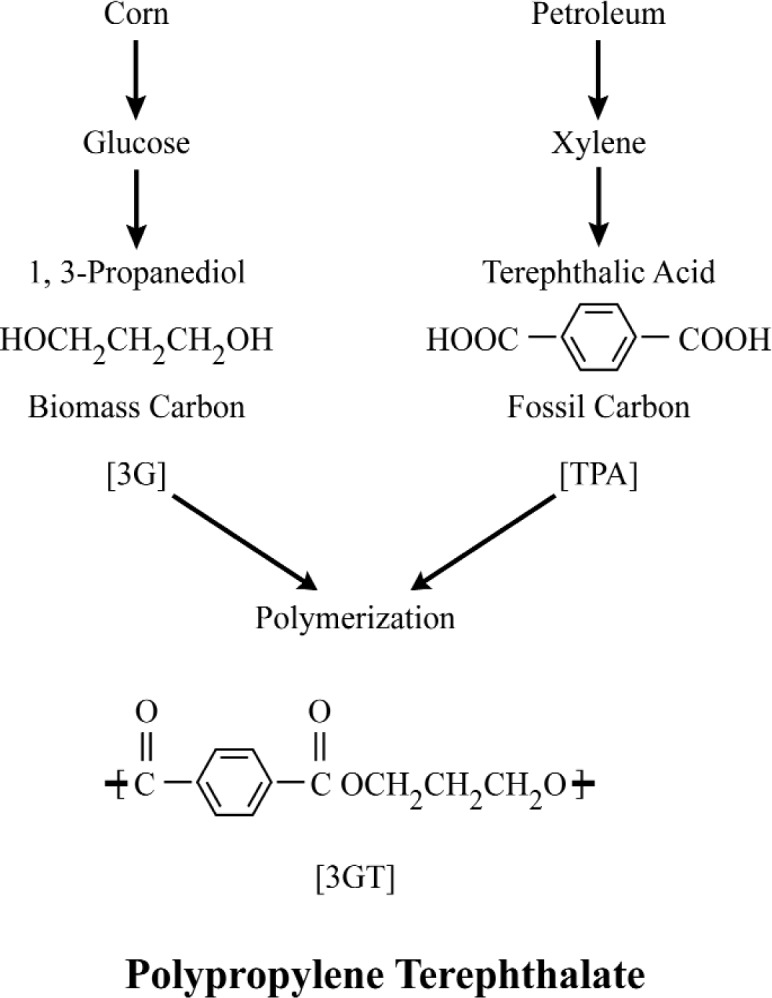
Polypropylene Terephthalate: biomass and fossil feedstocks. The 1,3, propanediol monomer is derived from a renewable (biomass) feedstock via laboratory biotechnology: conversion of glucose or cornstarch using a single microorganism. The copolymer has potential large volume demand, and is useful as a fiber, film, particle, and a molded article [25].

A case in point is the Cooperative Research and Development project between the NIST Chemical Science and Technology Laboratory and the DuPont Central Research and Development Laboratory [25]. The goal of the project was to demonstrate the capability to authenticate and date renewable (biosourced) feedstocks, chemical intermediates, and finished industrial products using high accuracy dual isotopic (^13^C–^14^C) “fingerprinting,” traceable to NIST. The specific project, as outlined in Fig. 9, was directed toward the unambiguous identification of the copolymer polypropylene terephthalate (3GT)) produced from the biosourced monomer 1,3-propanediol (3G), which was derived from corn as feedstock. (Terephthalic acid (TPA) served as the complementary monomer.) Isotopic discrimination was essential because it is not possible chemically to distinguish the biosourced 3G and 3GT from existing industrial materials that are fossil feedstock (petroleum) based. The ability to establish a unique isotopic fingerprint for the DuPont biotechnology materials was critical for the identification of the product as a unique composition of matter, and to track it in commerce. The work represents a frontier of high accuracy, dual isotope metrology, with ^13^C data *u*_r_< 0.01 %) serving to discriminate among different photosynthetic cycles, and ^14^C data (*u*_r_ < 0.5 %) serving both for quantitative fossil-biomass apportionment and for dating the year of growth of the biomass feedstock.

**Fig. 10:**
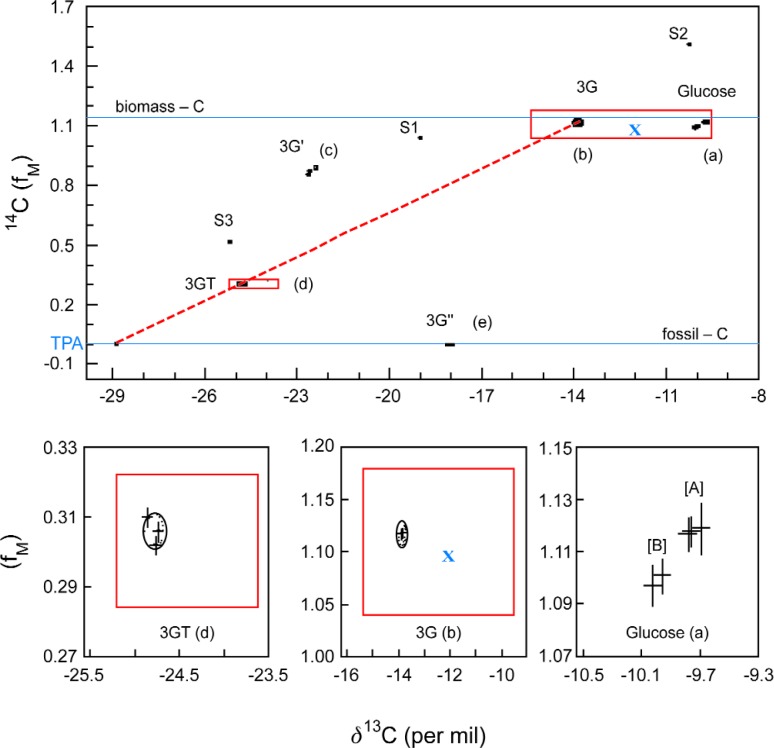
Unique Isotopic Signatures: the ^14^C-^13^C plane [25]. The main panel shows dual isotopic signatures f:or (1) NIST (S1, S3) and IAEA (S2) traceability standards, and (2) glucose from biomass (a), the new bio-sourced monomer 3G (b) (from cornstarch), the resulting copolymer 3GT (d), and pre-existing products 3G′, 3G″ (c, e). Expanded views of the authentication regions (red rectangles) for the copolymer (left) and monomer (center) are given in the bottom panels, plus ≈10-fold expansion (right) of the isotopic data for independent batches (A, B) of a biomass feedstock (glucose from corn). The blue “x” represents a blind (3G) validation sample.

**Fig. 11:**
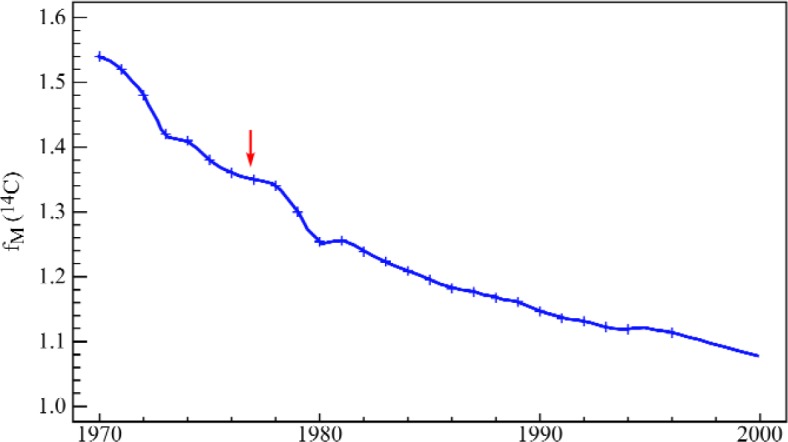
Short term ^14^C “decay” curve, representing geochemical relaxation of excess atmospheric ^14^C from nuclear testing [Levin et al., in (Ref. [19]; Ref. [20], Chap. 31). Information critical for the discussion in Sec. 7.2.1 is indicated by the arrow—namely, the sampling date and corresponding biomass ^14^C enrichment for SRM 1649a (urban dust).

A graphical summary of the results of the project is presented in Fig. 10, which shows the dual isotopic signatures of the copolymer (3GT) and bio-sourced monomer (3G); as well as values for isotopic reference materials (S1: SRM 4990B [oxalic acid]; S2: IAEA C6 [ANU sucrose]; S3: SRM 1649a [urban dust])., and pre-existing materials (3G′, 3G″). The dashed line joining the copolymer end members (3G, TPA) demonstrates *isotopic-stoichiometric mass balance.* Rectangular regions in red define the “scope of claims” (authentication regions) for the new isotopic compositions. The blue “x” in the figure represents data for an independent batch of the monomer—sent to NIST “blind” to test the validity of the authentication region for bio-sourced 3G. The results show both that the test was successful and that the separate production batches of the 3G monomer had unique isotopic signatures. The approximately ten-fold expansion of the isotopic data for two independent batches (A, B) of corn-glucose (bottom right) demonstrates the dual isotopic discrimination capability of the technique. In fact, using the short term “decay” curve of ^14^C (Fig. 11), it was possible to date the two batches to the nearest year of growth, 1994 (A) and 1996 (B), respectively. (Standard uncertainty bars shown.)

## 5. Anthropogenic Variations; “Trees Pollute”

The achievement of high precision, low background counting, discussed in Sec. 3, led also to the first isotopic evidence of global pollution with fossil CO_2_ —named the “Suess Effect,” after its discoverer. A dramatic monotonic drop in the ^14^C/^12^C ratio in tree rings beginning in the late 19th century, reflecting the use of coal during the Industrial Revolution, showed a 2.5 % fossil carbon dilution effect by the 1950s (Ref. [12], p. 289), after which it was eclipsed by the vast injection of “bomb” carbon. Thus began still another field of ^14^C science: the investigation of *anthropogenic variations*, particularly as related to environmental pollution.

### 5.1 Fossil-Biomass Carbon Source Apportionment

Research on more specific local or even regional carbonaceous pollution began slowly, because of the massive samples required. Heroic sampling efforts in the late 1950s demonstrated the principle by measurements of particulate carbon pollution in U.S. urban atmospheres [26, 27]. After a lapse of two decades, research in this area was renewed by the author, stimulated by a 1975 article in *Science* reporting that the culprit for a severe case of urban pollution in tidewater Virginia might be hydrocarbon emissions from trees [28]. The evidence was chemical and controvertible: plausible, but circumstantial evidence suggested that the air pollution was due to hydrocarbon emissions from trees rather from automobile exhaust or evaporation from nearby industrial and military storage tanks. The article concluded that “the relatively unsophisticated monitoring of [organic] pollutant concentrations … will rarely be of value in identifying [pollutant] sources …” Recognizing immediately that ^14^C could function as an undisputed discriminator, we decided to design miniature low level counters, capable of measuring just 10 mg carbon samples, more than two orders of magnitude smaller than those used in the two earlier studies. Apart from forest fires, we found that the trees were *not* the prime culprits, except for the case where humans were using the trees for fuel! A review of research in this area in the ensuing 20 years is given in Ref. [29].

**Fig. 12:**
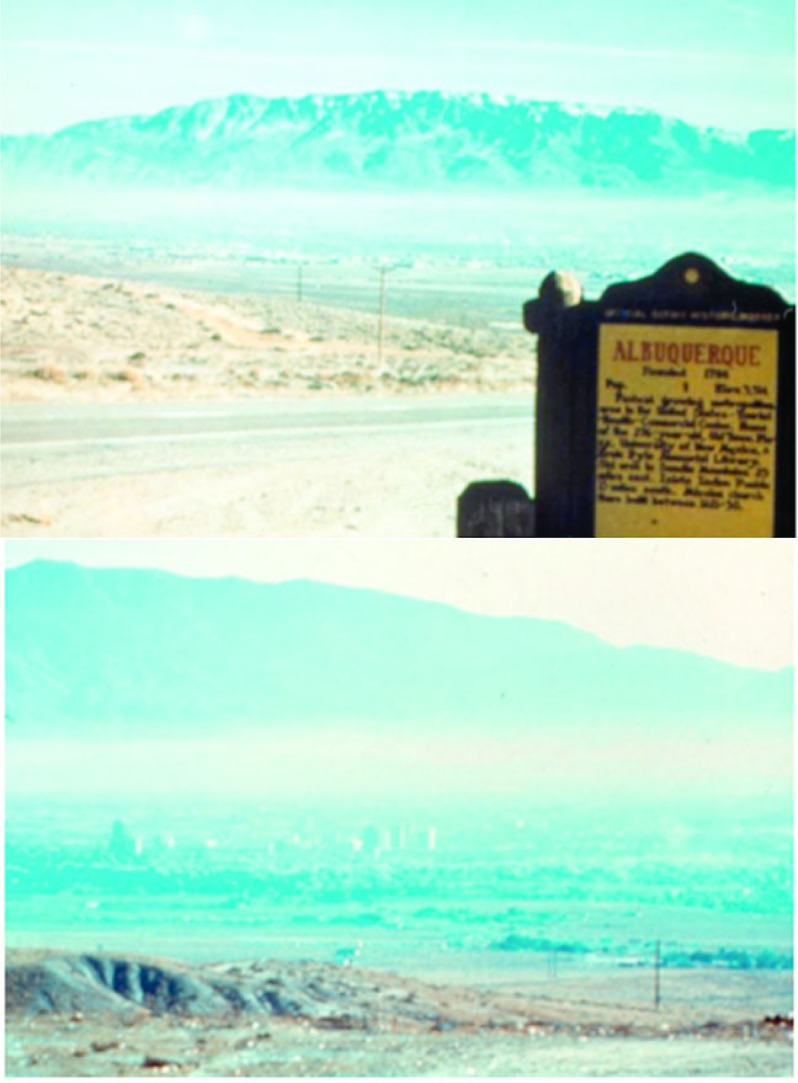
Anthropogenic ^14^C variations: fossil-biomass carbon apportionment of particulate air pollution in Albuquerque, New Mexico. (Photos showing visibility reduction in early morning (top) and mid-afternoon (bottom) are courtesy of R.K. Stevens [30].). ^14^C measurements quantified atmospheric soot from motor vehicles and residential wood-burning, and helped apportion concomitant data on particulate mutagenicity.

One illustration of ^14^C aerosol science is given in Fig. 12. It is drawn from perhaps the most extensive study to date of urban particulate pollution using ^14^C. The multi-year, multidisciplinary study of the origins of mutagenic aerosols in the atmospheres of several U.S. cities, focussed on Albuquerque, New Mexico during the winter of 1984–1985. The photos show the tremendous impact on visibility from particulate pollution from rush hour traffic. Results of the two month study of particulate carbon proved that daytime pollution (up to ≈ 65 %) was dominated by motor vehicle emissions (fossil carbon), and nighttime pollution (up to ≈ 95 %), by residential woodburning (biomass carbon), with the mutagenicity (potency) of the motor vehicle particles more severe by a factor of three [30]. Particulate carbon aerosols are now widely recognized as an extreme health hazard in a number of U.S. cities; and except for periods dominated by wildfires, major studies including ^14^C measurements have produced incontrovertible evidence that the urban episodes are dominated by fossil carbon, largely from motor vehicle exhaust [31].

Quantitative apportionment of natural and anthropogenic sources of particulate carbon, methane, carbon monoxide, and volatile organic ozone precursors in the atmosphere, meanwhile, has seen a significant expansion thanks to the sensitivity enhancement of accelerator mass spectrometry (AMS) [32, 33]. Most recently, with the emergence of micromolar ^14^C AMS, and GC/AMS, the ability to “date” individual chemical fractions in small samples is having important impacts on both artifact age accuracy, and our understanding of perturbations of the human and natural environments by fossil and biomass carbonaceous species. (See Section 7).

## 6. Accelerator Mass Spectrometry

### 6.1 The Invention

The second revolution in ^14^C measurement science was the discovery of a means to count ^14^C *atoms*, as opposed to ^14^C *decays* (beta particles). The potential impact on sensitivity was early recognized: inverting the first order nuclear decay relation, one finds that the ratio of the number of ^14^C atoms to the number of ^14^C decays for any given sample is simply (*τ/t*), where *τ* is the mean life (8270 a for ^14^C), and *t* is the counting time used for measurement of the disintegrations. Allowing for the difference in relative detection efficiency between AMS and low-level counting, and setting *t* to 2 d, gives a sensitivity enhancement of roughly 10^4^, in favor of AMS. This implies a dating capability of submilligram amounts of modern carbon.

The prize of radiocarbon dating at the milligram level was so great that major efforts were made to refine mass spectrometric techniques to render the 1.2 × 10^−12 14^C/^12^C ratio of modern carbon measurable; but, like Libby’s initial attempt to count natural radiocarbon (without enrichment), natural ^14^C proved unmeasurable by conventional mass spectrometry. Impediments from molecular ions and the extremely close isobar (^14^N: ∆*m/m* = 1.2 × 10^−5^) were overwhelming. Success came in 1977, however, when high energy (megavolt) nuclear accelerators were used as atomic ion mass spectrometers [34–36]. Two measurement ideas held the key: (1) Negative carbon ions are produced by a sputter ion source, using graphite as the target. (2) Following low energy mass selection, atomic and molecular negative ions are injected into an accelerator tube with a megavolt potential. The major isobar is eliminated because nitrogen does not form a stable negative ion. Passage of the high energy ions through a stripper gas or foil destroys all molecular ions through the “coulomb explosion,” leaving only atomic carbon ions in the +3 or +4 charge state. ^14^C/^12^C ratio measurements down to ca. 10^−15^ are thus made possible. Typical sample sizes are 0.5 mg to 1 mg; modern carbon yields 10 000 counts in just a few minutes; and instrument backgrounds are negligible (≤0.2 % modern, equivalent to a ^14^C age of ≥50 000 years BP).

**Fig. 13:**
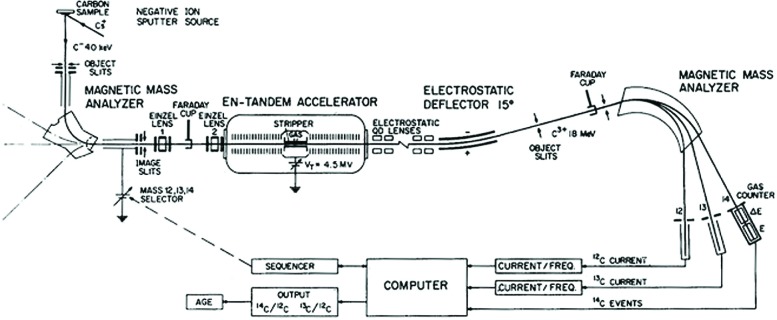
AMS: tandem accelerator at ETH, Zürich. Negative carbon ions, produced with a Cs^+^ sputter ion source, undergo low energy mass resolution and then are injected into the 4.5 MV accelerator tube. Molecular ions are destroyed by the stripper gas, and emerging 18 MeV C^+3^ beams of ^12^C, ^13^C, and ^14^C are mass analyzed and measured in current (stable C ions) and event (^14^C ions) detectors [37].

**Fig. 14:**
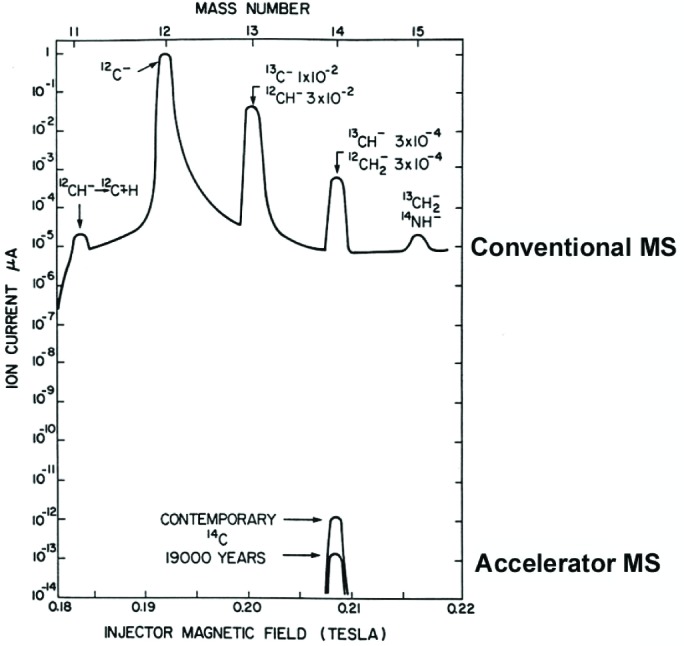
Conventional (top) vs accelerator (high energy) (bottom) mass spectrometry: ^14^C/^12^C sensitivity is enhanced by more than eight orders of magnitude through destruction of molecular ions (and unstable N^−^) (Ref. [20], Chap. 16).

A diagram of the accelerator at one of the leading facilities is given in Fig. 13 [37]. The dramatic impact of high energy (atomic ion) mass spectrometry is shown in Fig. 14, where it is clear that natural ^14^C is quite unmeasurable by low energy (conventional) mass spectrometry due to molecular ions exceeding the ^14^C signal by more than eight orders of magnitude (Ref. [20], Chap. 16]! Excellent reviews of the history, principles, and applications of AMS are given in Ref. [20] by H. Gove (Chap. 15) and R. Beukens (Chap. 16).

As noted in the reviews by Gove and Beukens, the AMS revolution has extended well beyond ^14^C, spawning a totally new research area in long-lived isotopic and ultra trace stable cosmo- and geo-chemistry and physics through its capability to measure ^3^H, ^14^C, ^26^Al, ^36^Cl, ^41^Ca, and ^129^I, and most recently, selected actinides.

Within one year of the publications announcing successful ^14^C AMS, another continuing series of international conferences was born. The first international AMS conference took place in 1978 in Rochester, New York. These conferences have continued on a triennial basis, with each proceedings occupying a special AMS conference issue of the journal, *Nuclear Instruments and Methods in Physics Research*.

### 6.2 The Shroud of Turin

**Fig. 15:**
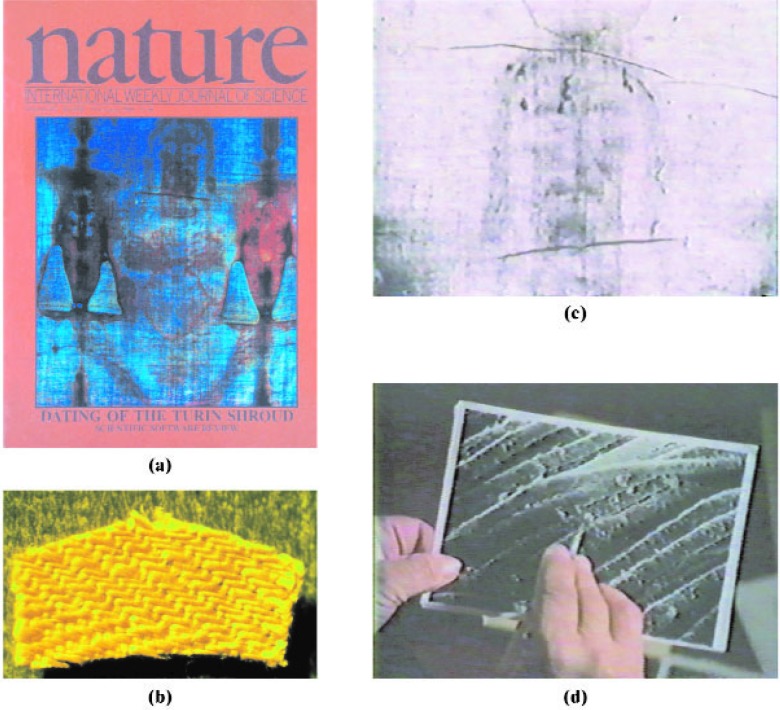
The Turin Shroud. Shown in the montage are: (15a, upper left), the cover of the issue of *Nature* (16 February 1989) reporting the results of the ^14^C measurements by AMS laboratories in Tucson, Zürich, and Oxford; and three singular features of the artifact: (15b, lower left), the ≈50 mg dating sample received by the Tucson laboratory, showing the distinctive weave (3:1 herringbone twill), with dimensions about 1 cm × 0.5 cm; (15c, upper right), the characteristic negative image, considered by some as a remarkable piece of mediaeval art; and (15d, lower right), a microphotograph by Max Frei showing individual fibers supporting pollen grains of presumed unique origin [38, 39].

The radiocarbon dating of the Turin Shroud is arguably the best known dating application of accelerator mass spectrometry, at least to the lay public. It could not, or at least it would not have taken place without AMS, because most decay (beta) counting techniques would have consumed a significant fraction of this artifact. Although still a destructive analytical technique, AMS required only “a postage stamp” amount of the linen cloth (Ref. [20], Chap. 15). This particular exercise is having a metrological impact well beyond the radiocarbon date, *per se.* This is shown, in part, by widely accepted statements (1) concerning scientific investigations of the Shroud, and (2) following publication of the *Nature* article announcing radiocarbon dating results (Fig. 15; Ref. [38]).

1. “The Shroud of Turin is the single, most studied artifact in human history.”
2. “The *Nature* (^14^C) article has had more impact on Shroud research than any other paper ever written on the subject.”

The article, which was prepared by three of the most prestigious AMS laboratories, is available to the general public on the web (*www.shroud.com/nature.htm*). Together with public television [39], it is helping to create a broad awareness and understanding of the nature and importance of the AMS measurement capability. Secondly, because of controversy surrounding the meaning of the radiocarbon result, measurement aspects of artifact dating have been given intense scrutiny. Such scrutiny is quite positive, for it gives the possibility of added insight into unsuspected phenomena and sources of measurement uncertainty.

The Turin Shroud is believed by many to be the burial cloth of Christ. The documented record, however, goes back only to the Middle Ages, to Lirey, France (ca. 1353 AD) with the first firm date being 1357 AD when it was displayed in a Lirey church. Radiocarbon dating was seen immediately as a definitive method to decide whether the “Lirey Shroud” could have come from flax grown in the 1st century AD. The Shroud image, considered by some to be the skilled work of a mediaeval artist, shows a full length image of a crucified man; but as a *negative image* [Fig. 15c].^9^ Prior to the AMS measurements, the Shroud was subject to intensive examination by photography, spectroscopy, art and textile analysis, and palynology [38–40]. The unique herringbone twill [Fig. 15b] is considered consistent with a 1st Century date; and pollen grains found on the cloth [Fig. 15d] are stated by Max Frei to have originated from a plant found only in the region of Jerusalem. Radiocarbon dating of the cloth, however, yielded a result of 1262 to 1384 AD (95 % confidence interval) [38].

**Fig. 16:**
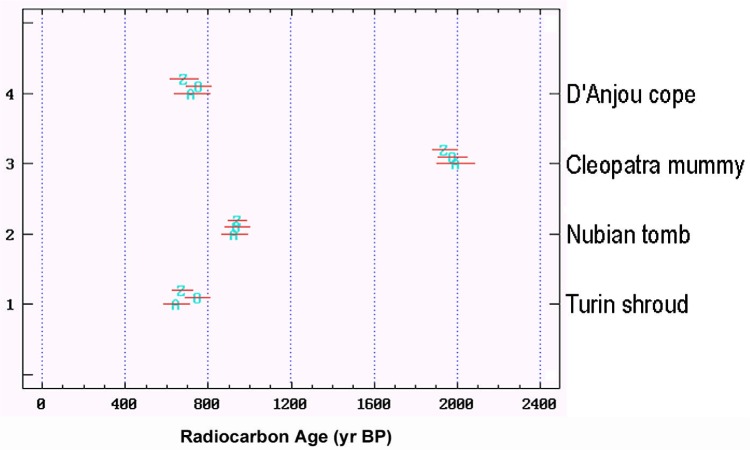
AMS ^14^C dating results (“blind”) for the Turin Shroud (sample-1) and three control samples of known age (samples-2,3,4), from the three AMS laboratories: Z (Zürich), O (Oxford), and A (Arizona). Dates are expressed as “Radiocarbon Years” before present (BP); uncertainties represent 95 % confidence intervals [38].

**Fig. 17:**
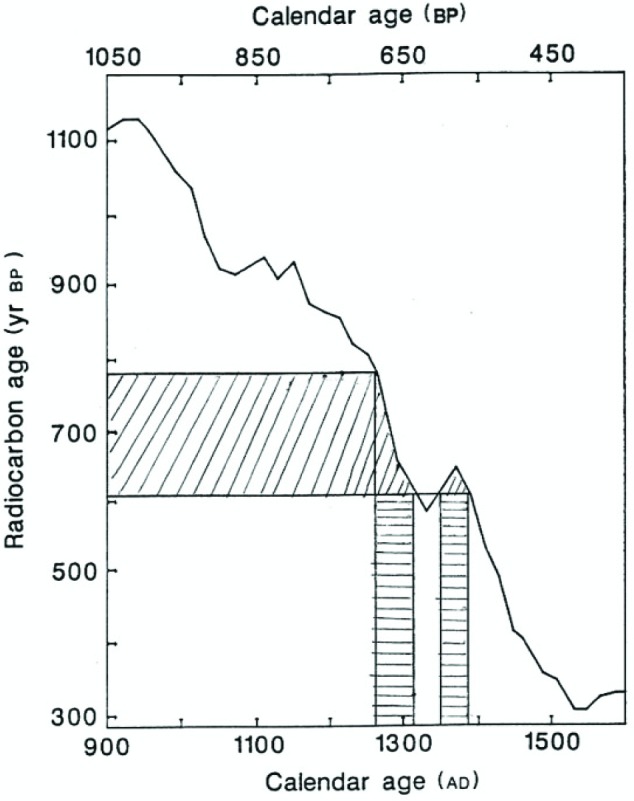
Transformation of the Radiocarbon Age (BP) to the Calendar Age (AD) of the Shroud. The ^14^C age (95 % CI) of (691 ± 31) BP corresponds to a two-valued calendar age as a result of the non-monotonic radiocarbon dating calibration curve. As indicated in the figure, the projected calendar age ranges are: (1262–1312) AD and (1353–1384) AD [38].

Apart from sampling,^10^ the AMS measurements were performed taking the strictest quality control measures. Three highly competent laboratories were selected: the University of Arizona, Oxford University, and the Swiss Federal Institute of Technology [ETH] in Zürich. Samples of the Shroud, plus three control samples of known age, were distributed blind to the three laboratories. Control of this operation (distribution of samples, collection of results) was the responsibility of Michael Tite of the British Museum. The accuracy and precision of the interlaboratory data for the control samples were outstanding, leaving no doubt as to the quality of the AMS measurement technique (Fig. 16). Sample-1 (Shroud) results, however, were just marginally consistent among the three laboratories, prompting the authors of Ref. [38] to state that “it is unlikely that the errors quoted by the laboratories for sample-1 fully reflect the overall scatter.” Consistent with the discussion in Sec. 2, the ^14^C age measurements are reported in “^14^C years BP.” Transformation of these ages to calendar ages must take into account the natural ^14^C variations, using the dendrochronological calibration curve [13]. The transformation is shown in Fig. 17, which demonstrates also an interesting aspect of the non-monotonic calibration function: namely, exclusion of the period between 1312 AD and 1353 AD from the 95 % confidence interval. In addition, an interesting link exists between this figure and Fig. 6 (Maunder Minimum), in that the same solar-activity-induced ^14^C variations are represented. A comparison of the two figures shows that the radiocarbon date (691 BP), near the end of a significant calibration curve protrusion (Fig. 17), corresponds to the end of the 13th century warm period having high solar activity (Fig. 6).

Consistency of the AMS results with the existing (Lirey) documentation seems compelling, but a wave of questioning has followed—not of the AMS method, but of possible artifacts that could have affected the linen and invalidated the ^14^C result (Ref. [40], Chap. 1, Refs. [41], [42]). A sampling of the creative hypotheses put forward is given in Table 2. The first, for example, is based on the premise that nuclear reactions involving the substantial amount of deuterium contained in a human body could produce neutrons, which might then produce excess ^14^C through the (n,p) reaction, making the age too young. The proposed deuteron reactions, however, are either qualitatively or quantitatively inaccurate—barring an unnatural burst of high energy photons (photofission). The third proposal raises the question of non-contemporaneous organic matter— whether from incompletely removed carbon contamination from “oil, wax, tears, and smoke” that the cloth had been exposed to, or from bacterial attack and deposit over the ages. Apart from the effects of such factors on the Shroud, the issue of organic reactions and non-contemporaneous contamination of ancient materials can be a very serious and complex matter, deserving quantitative investigation of the possible impacts on measurement accuracy.^10^ Research questions of this sort, including the classic problem of dating ancient bone, form one of the key stimuli for the development of “molecular dating”—the topic of the following section.

## 7. Emergence of µ-Molar ^14^C Metrology

Radiocarbon metrology is at the very moment in the midst of still another revolution, involving the dating (or isotopic speciation) of pure chemical fractions: “molecular dating.” For trace species, such as polycyclic aromatic hydrocarbons (PAHs), or remote, low concentration samples, such as the soot or pollen in the free troposphere or in ice cores, the sensitivity of AMS is challenged to its ultimate. In order to understand the nature of the challenge it is interesting to consider the limiting factors. In a recent study it was shown that 10 % Poisson “error” (standard uncertainty) can be achieved with 0.9 µg modern carbon, whereas machine background is equivalent to 0.2 µg or less [43]. Sample processing blanks, however, may range from 1 µg to 15 µg or more, and they may consist of both biomass carbon and fossil carbon [44]. Thus, the ultimate limiting factor for very small sample AMS is the overall isotopic-chemical blank. Environmental studies of ^14^C in individual chemical compounds can be successful at the 1 µg to 10 µg level, but only with stringent control of the variability of the blank. This is in sharp contrast with small sample, low-level counting where the Poisson modern carbon limit (ca. 3 mg) and background limit (ca. 5 mg equivalent) far exceed the typical sample preparation blank (ca. 40 µg) [29].^11^

Some illustrations of pure compound “dating” by NIST and collaborators are given in Table 3. The first item refers to the aforementioned 1 µg capability, using “dilution AMS.” For thermally stable species such as soot and pollen, we have the possibility of controlling the sample preparation blank to less than 0.2 µg by applying a “thermal discriminator” at a critical stage of the process. Microgram level ^14^C soot studies have already been successful in Greenland snow; and pollen studies hold great promise for ice core dating, and perhaps even for dating the pollen found by Max Frei on the Turin Shroud.^12^ An important measurement issue for ice core pollen relates to the amount needed for a given dating precision. To give a rough estimate: assuming 50 ng carbon per pollen grain, a pollen age of 2000 years, and 5 % Poisson imprecision (σ≈400 years); one would need to collect about 100 pollen grains. This might be accomplished in a few hours, using the “hand picking” microscope technique of Long et al. [48].

### 7.1 Long-Range Transport of Fossil and Biomass Aerosol

**Fig. 18:**
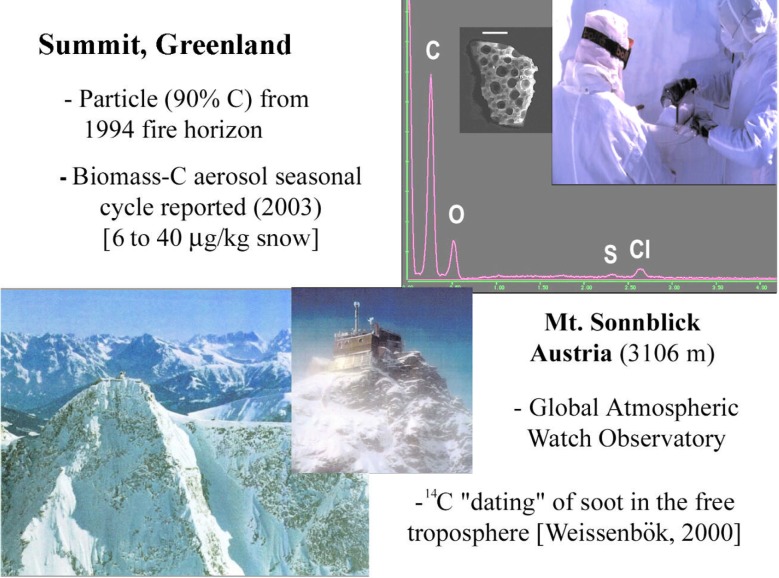
Submicromolar ^14^C apportionment of anthropogenic and natural carbonaceous aerosols at remote sites in Europe and Greenland provides knowledge of their impacts on present and paleoclimate [49–51].

Ongoing multidisciplinary, multi-institutional research on soot particles in remote and paleo-atmospheres, which is absolutely dependent on the small sample dating capability, is indicated in Fig. 18. The upper portion of the figure relates to climate oriented research on the sources and transport of fossil and biomass aerosol to the remote Arctic [49]; the lower portion relates to atmospheric and paleoatmospheric research at Alpine high altitude stations and ice cores [50,51]. In the remainder of this section we present some of the highlights and measurement challenges of the first project, on the long-range transport of carbonaceous particles to Summit, Greenland.

**Fig. 19:**
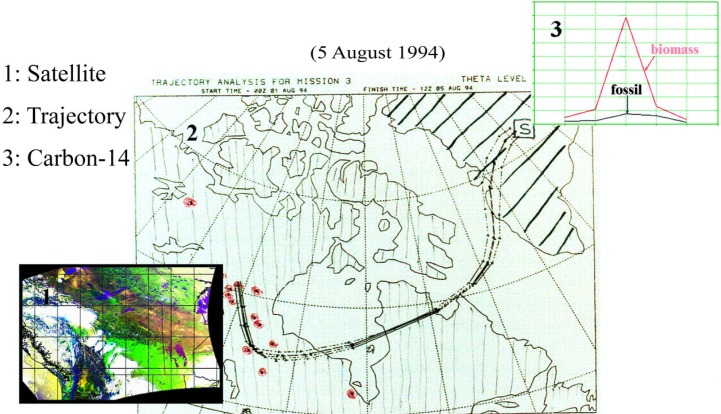
Massive (6 d, 3000 km) transport of soot from boreal wildfires in Canada to Summit, Greenland. Left inset [1]: AVHRR satellite image of wildfire region [29]; Center [2]: 6 d backtrajectories [52]; Right inset [3]: seven-fold,1 d increase in biomass-C (upper, red curve) at Summit [S] on 5 Aug 1994; fossil-C (lower, black curve), was little changed [49, 29]).

**Fig. 20:**
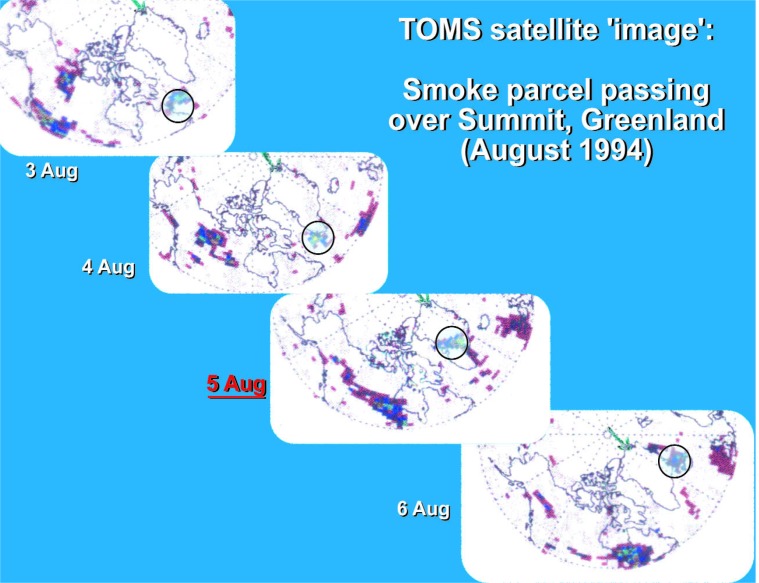
Direct, time lapse observation of the track of the August 1994 smoke parcel by TOMS (differential UV) satellite imagery [49]. Consistent with the ^14^C (biomass carbon) data, the cloud of smoke, indicated by the light turquoise circles, is present over central Greenland for 1 day only, 5 August 1994.

Cooperative research on this project, between NIST and the Climate Change Research Center at the University of New Hampshire (UNH), began in 1994. It was catalyzed by the discovery of an unusually heavy loading of soot on one of the air filters used for ^7^Be sampling at Summit, Greenland by Jack Dibb of UNH [52]. The Summit soot had been ascribed to the combination of intense boreal wildfire activity in the lower Hudson’s Bay region of Canada and exceptional atmospheric transport to central Greenland. Measurement of ^14^C in the filter sample yielded definitive evidence for biomass burning as the source of the soot. On one day only (5 August 1994), the biomass carbon increased by nearly an order of magnitude, with scarcely any change in the fossil carbon concentration on the filter. Supporting data for the origin of the biomass burning carbon came from backtrajectory analysis, AVHRR (infrared) satellite imagery of the source region, and TOMS (ultraviolet) satellite imagery that was able to chart the course of the soot particles from the source wildfires to Summit. The several parts of this remarkable event are assembled in Figs. 19, 20 [29,49,52].^13^

Since snow and ice can serve as natural archives for atmospheric events, one may expect to find chemical evidence of prior years’ fire seasons in snowpits, firn, and ultimately ice cores. This is illustrated in the upper right portion of Fig. 18, which shows depth profile sampling in a snowpit at Summit, overlaying an energy dispersive spectrum and SEM image of a char particle found near the 1994 fire horizon in a 1996 snowpit [29]. An organic tracer of conifer combustion, methyl dehydroabietate, was found also at the same depth [53].

**Fig. 21:**
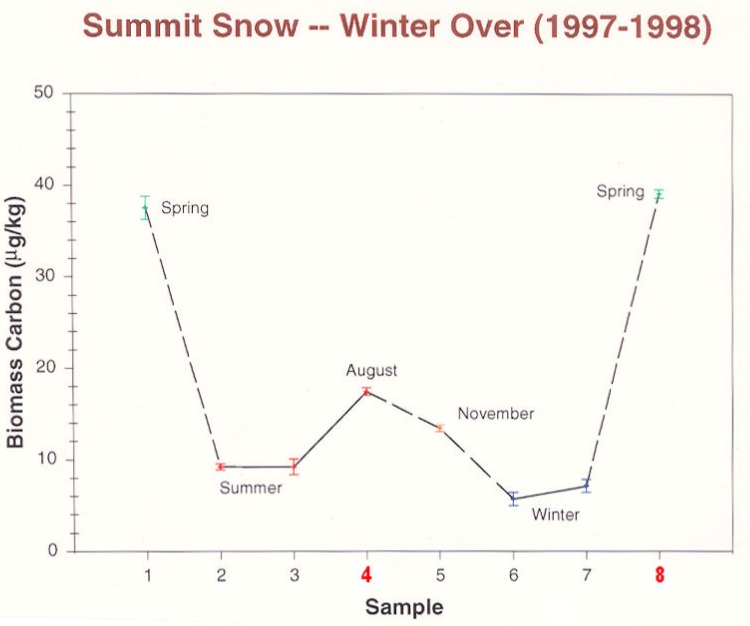
First evidence of a seasonal pattern in biomass carbon aerosol in surface snow in central Greenland [55, 56]. Fundamental differences were found between the biomass carbon peaks in summer (sample-4 [WO4]), and spring (sample-8 [WO8]) via “multi-spectroscopic” macro- and micro-chemical analysis.

**Fig. 22:**
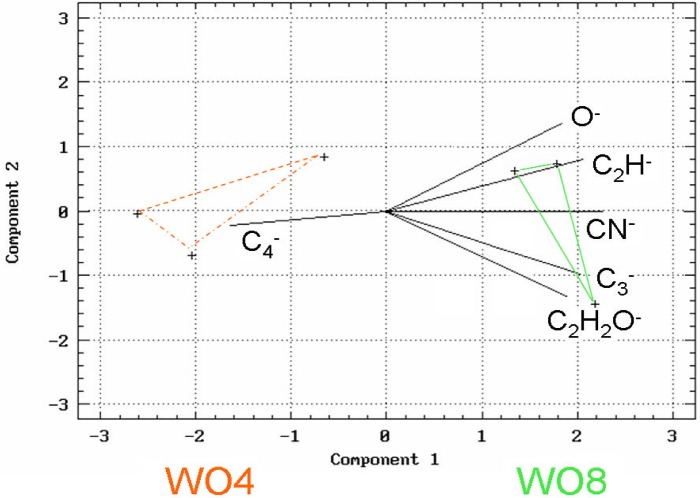
PCA biplot of laser microprobe mass spectral data; compositional contrast between particles from the summer biomass peak (WO4, red: C*_n_*^−^ cluster ions favored) and the spring biomass peak (WO8, green: oxygenates favored) [55].

Atmospheric science entered a new phase at Summit during the “Winter-Over” project (1997–1998) [54]. For the first time, direct sampling of air and surface snow took place over the polar winter, extending from June 1997 to April 1998. A special achievement of micro-molar ^14^C “dating” was the first seasonal data for carbonaceous particles, deposited with the surface snow.^14^ The seasonal record for biomass carbon particles, shown in Fig. 21, was striking [55]. The large spring peaks, in particular, consisted primarily of biomass carbon: 0.76 (*u* = 0.03) modern carbon mass fraction (*f*_M_) for sample-1 (WO1), and 0.94 (0.01) mass fraction for sample-8 (WO8). Beyond the fossil-biomass apportionment, however, lay questions about the nature and origin of the carbonaceous aerosol. Especially intriguing are contrasts between the samples showing summer [sample-4 (WO4)] and spring [sample-8 (WO8)] biomass-C maxima in Fig. 21. To explore these, a “multi-spectroscopic” approach was taken, through which insights and supporting evidence were derived from a variety of analytical techniques. Results for one of the microanalytical techniques employed, laser microprobe mass spectrometry (LAMMS), are shown in Fig. 22.^15^ The figure uses a principal component projection to summarize multivariate (multi-mass) contrasts between the summer and spring biomass peaks. It shows that the three summer (WO4) sub-samples tend to favor C*_n_*^−^ cluster ions (n-even), typical of condensed carbon structure (and graphite), whereas the three spring (WO8) sub-samples exhibit a more complex, oxygenated structure such as occurs with biopolymers.

Findings from other techniques:

- *Thermal-optical analysis.* Distinctive seasonal volatilization/decomposition patterns were seen as samples were heated in a stream on helium. The summer sample (WO4) had a predominant high temperature peak at ≈560 °C and little evidence of charring (4 %), whereas the spring sample (WO8) had a predominant peak at ≈410 °C and major charring (19 %). Thermal analysis of a powdered wood (oak) reference material showed a thermal peak at the approximately same temperature as WO8, with 21 % charring, implying the presence of a major cellulosic component in this sample.
- *Ion chromatography.* Fire tracers (NH_4_^+^, K^+^) accompanied WO4; soil tracers (Ca^++^, Mg^++^) accompanied WO8
- *Backtrajectories.* For WO4, strong transport was indicated from regions of annual wildfires in the Canadian Northwest; for WO8, strong transport was indicated from the agricultural regions of the upper Midwest—both representing transport distances of some 8 Mm.
- *Electron probe microanalysis.* For WO4, up to 90 % C (mass fraction) was observed in individual, µm size particles, with C > O for the most abundant (core) particles; for WO8, maximum C particles had a C:O ratio consistent with cellulosic biopolymer, and C < O for the core particles.

The weight of multi-spectroscopic evidence thus indicates that the summer (WO4) and spring (WO8) biomass particles *do not represent the same type of biomass.* Rather, the WO4 particles appear to include a soot component from high temperature combustion (motor vehicles, wildfires). The WO8 particles, whose carbon derives *almost* entirely from biomass, appear to have a major biopolymer component, such as cellulose and other bio-materials associated with soil and vegetative carbon. These findings are consistent with work by Puxbaum and colleagues, who have found by direct chemical analysis, significant amounts of cellulose, bacteria, and fungal spores in atmospheric particles [58, 59].

### 7.2 Isotopic Speciation in Ancient Bones and Contemporary Particles

**Fig. 23:**
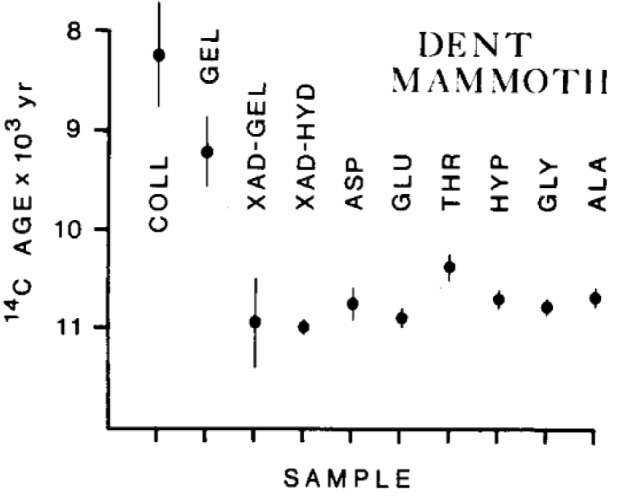
“Molecular Dating” of individual amino acids in ancient bone. Radiocarbon ages of commonly dated (collagen, gelatin) fractions were 2000 to 3000 years too young as a result of environmental degradation; pure molecular fractions (amino acids) were self-consistent and in agreement with the Clovis culture age [60].

The dating of ancient bones has been notably unreliable because of diagenesis and isotopic contamination that occur with millennia of environmental exposure. Molecular dating of individual amino acids in such bones has proven to be one of the most effective means to overcome this problem. Figure 23 shows dramatically how the apparent radiocarbon age of the Dent Mammoth changed from ca. 8000 BP to ca. 11 000 BP, as the dated chemical fraction was refined from the crude collagen fraction to the individual amino acids. The known radiocarbon age is given as ≈11,000 BP, based on association with Clovis culture artifacts, and biostratigraphy [60]. (Note that the “calibrated” or corrected calendar age, derived from the radiocarbon calibration curve [13], is roughly 1500 years older than the radiocarbon age for this time period.) The commonly dated organic fractions from bones (weak acid insoluble collagen [COLL] and gelatin [GEL]) gave ages that were at odds with the archaeological evidence—suggesting recent humate contamination. When the diagenesis-resistant molecular components were isolated (individual amino acids and the collagen hydrolysates [XAD-HYD]), age concordance among the individual amino acids and with the archaeological evidence indicated reliability. Had contamination from bio-intrusive material having a different chemical (amino acid) pattern occurred, amino acid age heterogeneity would have been expected [60]. This work could not have been accomplished without the ability to date 80 µg carbon fractions.

An historical footnote related to this work involves the question of the ancestors of the North American Clovis culture. Since the Clovis sites give the earliest unequivocal data on the “peopling” of the Americas, it has been of enormous interest to find a geochronological link to an earlier culture. The most popular belief that the Clovis progenitors had arrived over the “Bering Land Bridge” from Siberia has recently been put into doubt, however, with new ^14^C evidence that one of the most likely pre-Clovis sites in northeastern Siberia is 4000 years younger than previously believed. Dating at ≈13 000 calendar years ago, it is doubtful that migration could have transpired quickly enough to give rise to the Clovis culture (13 600 to 12 600 calendar years BP) in the North American Southwest [61].

#### 7.2.1 Urban Dust (SRM 1649a): a Unique Isotopic-Molecular Reference Material

**Fig. 24:**
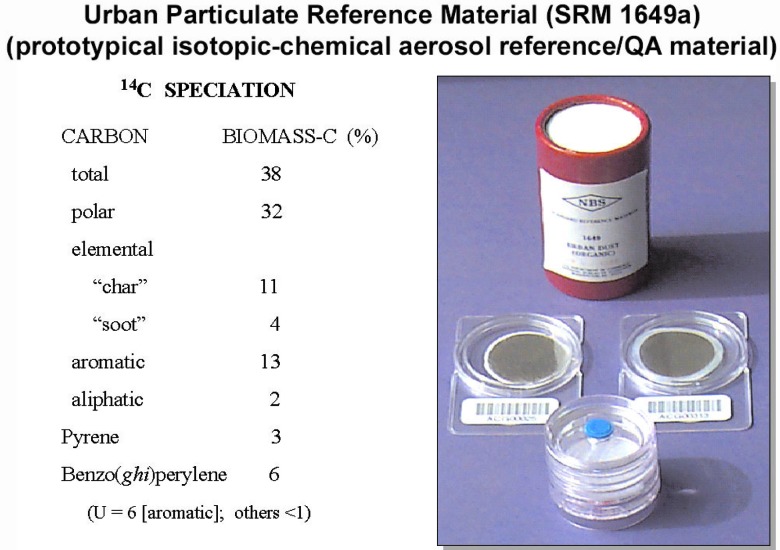
NIST Standard Reference Material 1649a (“urban dust”). Photograph of the bulk reference material and derived “filter samples” for QA of atmospheric elemental carbon (EC). ^14^C data listed indicate the mass fraction (%) of biomass-C in the several chemical fractions [29, 62].

SRM 1649a is NIST’s most highly characterized natural matrix Standard Reference Material, and it is the *only one* for which there are certificate values for ^14^C in individual chemical fractions and pure molecular species. The “carbon” portion of the Certificate of Analysis was developed through an extensive international interlaboratory comparison, involving eighteen teams of analytical experts from eleven institutions [62]. The particle-based SRM, which has been characterized for nearly 200 chemical species and properties, serves as an essential quality assurance material for a remarkably broad range of disciplines, from the monitoring of pesticides, PCBs, and particulate mutagenic activity to basic organic geochemistry to isotopic apportionment of carbonaceous particles. A dramatic illustration of the ^14^C isotopic heterogeneity in this reference material is given in Fig. 24. The biomass carbon mass fractions are seen to range from about 2 % (aliphatic extract) to 38 % (total carbon). Thus, the aliphatic fraction derives essentially (≈98 %) from fossil fuel emissions, and, on average, fossil sources account for some 60 % of the carbon in these particles.

Note that the Certificate of Analysis [63] provides ^14^C data expressed in the proper reference units as fraction of modern carbon (*f*_M_). To emphasize the more meaningful fossil-biomass carbon source dichotomy, however, we have chosen to present the information here in terms of the fraction of biomass carbon. Conversion is based on the “post-bomb” enrichment of ^14^C in the living biosphere, as shown in Fig. 11. Sampling for SRM 1649a took place in 1976–1977; the enrichment factor for biomass carbon at that time, indicated by the red arrow in the figure, was 1.35.

One of the most important outcomes of the SRM 1649a intercomparison exercise was the set of data obtained for “elemental carbon” (EC). EC (sometimes known as “black carbon”) is routinely monitored in urban and rural aerosols, and it is of major concern because of its presumed impacts on health, visibility, and climate (radiation absorption). SRM 1649a potentially can serve as a key laboratory quality assurance reference material for EC measurement. Results of the largest intercomparison to date of EC in a uniform reference material, however, indicate a severe measurement problem: relative values for the reported data span a range of 7.5, showing very significant method dependence. Three clusters of results for the mass fraction of EC (relative to total-C), reported as *information values* on the Certificate of Analysis, are 0.075, 0.28, and 0.46. (For the ^14^C data in Fig. 24, cluster-1 EC has been labeled “soot” and cluster-3 EC, “char.” ^14^C was not determined in cluster-2 EC.) The fundamental problem is that EC is *not a pure substance*, so a unique “true value” for EC may not exist, in principle.^16^ Some interesting insights into the meaning of certain of the EC results follow, however, from the ^14^C EC speciation data.

##### Isotopic consistency

Measurement of ^14^C in multiple chemical fractions offers the possibility of two very interesting and important consistency tests: (1) assessment of *isotopic-chemical consistency* among chemically-related fractions, and (2) assessment of overall *isotopic-mass balance.* The first test is illustrated by comparison of the ^14^C content of the EC fraction with that of the PAH fraction (on average). To the extent that both components originate from the same source, acetylenic free radicals that generate polyaromatic structures in the flaming stage of combustion, one would expect similar ^14^C composition. Such is the case for ^14^C in cluster-1 EC (labeled “soot” in Fig. 24), but not for cluster-3 EC (labeled “char”). The lack of isotopic consistency for cluster-3 EC is the stimulus for the different label, since this manifestation of EC necessarily reflects a different mix of fossil-biomass sources than the flaming stage EC, which derives primarily from fossil fuel carbon.

Regarding the second test, the ^14^C data in Fig. 24 demonstrate that isotopic-mass balance cannot be achieved with the current isotopic-chemical data. Since the biomass carbon fraction on average (38 % mass fraction) exceeds that of *all* other measured fractions, there *must be a significant missing biomass carbon component*. This matter is addressed in [62], where it is suggested that unmeasured biopolymers may account for more than 45 % of the residual (non-extractable, non-EC) carbon mass. Cellulose is one excellent candidate [58].

##### GC/AMS

**Fig. 25:**
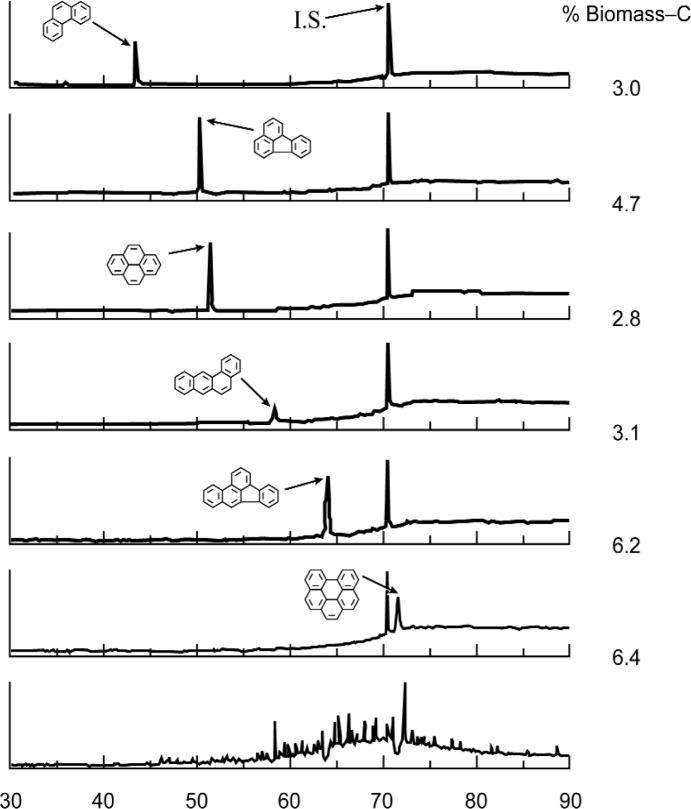
Gas chromatography/accelerator mass spectrometry (GC/AMS): AMS following automated prep-scale capillary GC yields “dates” (equivalent biomass carbon mass fractions) for micromolar amounts of individual polycyclic aromatic hydrocarbons [63–65]. (Results shown for NIST SRM 1649a; “I.S.” denotes an internal standard; abscissa indicates retention time (min).)

Finally, the “molecular dating” of individual PAH in SRM 1649a epitomizes one of the latest advances in micromolar ^14^C measurement science: the capability to link chromatographic isolation of pure chemical compounds to AMS determination of ^14^C^/12^C. Results of applying off-line GC/AMS to six PAHs recovered from the aromatic fraction of SRM 1649a are shown in Fig. 25. The critical first step was the sequential isolation of tens of micrograms of the six PAHs in separate traps by automated preparative scale capillary gas chromatography [66]. The individually trapped PAHs were then oxidized and converted to AMS targets. These results represent the first such data ever available for an atmospheric particulate SRM, and although such compounds are only trace constituents of atmospheric particles (≈10 µg/g), they are of great consequence due to their mutagenic and carcinogenic properties. In this case, as shown in Fig. 25, radiocarbon dating of the individual PAHs revealed these congeners to be isotopically heterogeneous, and demonstrated a basic flaw in the conventional wisdom that the heavier PAHs, in particular, are more likely to be produced strictly from fossil fuel combustion sources.

On-line GC/AMS is nearly upon us. The linkage of gas (or liquid) chromatographic separation, and *direct injection* of microgram amounts of pure compounds into the ion source of an accelerator mass spectrometer, is under active investigation in several AMS laboratories; and it promises a new dimension in the practice of radiocarbon dating at the molecular level that may have an impact on archaeology and isotopic biogeochemistry comparable to that of GC/MS on analytical, physical, organic, and biochemistry [67].

## 8. Epilogue

Libby’s discovery, and the remarkable developments that followed, arose from a scientific question (freely translated): “What will become of the cosmic ray neutrons?” It is noteworthy that an “academic son” of this Nobel Laureate also posed a scientific question to himself. F. Sherwood Rowland’s question also led to an unexpected discovery having major practical import for mankind: the possible destruction of the stratospheric ozone layer. Rowland’s query, also culminating in a Nobel Prize (1995), was “I began to wonder what was going to happen to this man-made compound [trichlorofluoromethane] newly introduced into the atmosphere” [68].

May this historical journey into scientific discovery, as an outgrowth of seemingly simple scientific curiosity, and the consequent unanticipated scientific-metrological revolutions, encourage students to examine the original historical literature documenting such discoveries, and to realize that profound unforeseen developments may be in store for a presumably “mature” scientific discipline.

## Figures and Tables

**Table 1:** Libby’s Measurement Challenge

• Cosmic ray neutron intensity: 2 n cm^−2^ s^−1^
• Exchangeable carbon reservoir: 8.5 g cm^−2^
• Estimated ^14^C activity: 14 dpm g^−1^ (0.23 Bq g^−1^)
• Sample size (detector efficiency): 8 g carbon (5.5 %)
• Estimated modern carbon rate 6.2 cpm (min^−1^)
• Background rate: 500 cpm (unshielded), 100 cpm (20 cm Fe)
Assumptions:
Constant production rate
Fixed exchangeable C reservoir (uniform distribution)

**Table 2:** Creative Hypotheses

• Excess ^14^C from deuterium spontaneous fission; cold fusion
• ^14^C isotopic fractionation/exchange (fire of 1532 AD biased sampling; “age” depends on location
• Bioplastic coating; non-contemporaneous with linen pretreatment chemistry

**Table 3:** Molecular Dating (^14^C AMS at the microgram level)

• *Dilution AMS* quantifies 0.9 µg modern carbon (1999)
– soot/pollen blank controllable to ~0.2 µg (σ ≈ 60 ng)
– challenge: dating pure pollen grains from the Shroud
• *Fossil and biomass aerosol sources* characterized in remote atmosphere/cryosphere (2.9 µg biomass soot quantified)
• *Individual amino acids* dated in mammoth bones (LC/AMS)
• *Individual polycyclic aromatic hydrocarbons* dated in atmospheric particles and marine sediment (GC/AMS)

## Footnotes

1 Figure 1 shows Libby as the author first met him, shortly after the latter entered the University of Chicago as a graduate student in chemistry.

2 Production rate and reservoir parameters are taken from the Nobel lecture [4]; these values differ somewhat from those used by Libby in [5] and in the first edition of his book [2]. The half-life (5568 a) is the “Libby half-life” which by convention is used to calculate “radiocarbon ages;” the current accepted value for the physical half-life is (5730 ± 40) a [5a].

3 To fully appreciate the nature of the experimental impediments and flashes of insight along the path to discovery, students are encouraged to study the original scientific literature, as given here, rather than restricting attention to subsequent summaries in textbooks.

4 The neutron intensity in the atmosphere, and hence the ^14^C production profile, has major variations vertically (because of cosmic ray absorption with atmospheric depth) and latitudinally (because of geomagnetic shielding)—See Figs. 2 and 3 in Ref. [2]. Because ^14^C has such a long mean life (≈8000 a), however, it was expected that any residual gradients in the global exchange reservoir would be undetectable, given the 3 % to 5 % uncertainties of Libby’s original measurements (Ref. [2], Chap. I).

5 Several secondary standards for ^14^C dating are available through the International Atomic Energy Agency. These materials, designated IAEA C1 – C8, consist of wood, cellulose, sucrose, and carbonate; they cover a range of 0.00 pMC to 150.6 pMC, and have been subject to an international comparison [11]. Note that pMC (percent modern carbon) refers to *f*_M_ expressed as a percentage.

6 The deciding factor for high precision ^14^C measurement was the successful development of CO_2_ gas proportional counting, after several failed attempts. Compared to Libby’s solid sample (graphite) technique, the CO_2_ method resulted in smaller sample sizes and efficiency enhancement by nearly a factor of twenty.

7 The relatively imprecise dendro-calibration curve in Fig. 5 extends to ca. 5000 BC. Meanwhile, the radiocarbon dating calibration function has undergone considerable refinement: it now comprises an extensive database, and it has become an essential element of *all* radiocarbon dating. The 1986 Calibration Issue of the journal *Radiocarbon* [13] has a compilation going back to ca. 8000 BC. More recent attempts at extending the record much further back in time have utilized ^14^C comparisons with other dating methods, notably U/Th disequilibrium dating. By this means, calibration data have been given for periods beyond 20 000 BC [14].

8 The advent of accelerator mass spectrometry, as discussed in Sec. 6 of this article, has given a major boost to our knowledge of ocean circulation. Information gained through the GEOSECS program has been greatly amplified in the World Ocean Circulation Experiment (WOCE), where requisite sample sizes were reduced from 200 L of sea water each, to less than 1 L; and the ^14^C ocean circulation database grew by more than 10 000 dates during the 1990s [23].

9 Figure 15c and 15d images are from the documentary prepared by the British Broadcasting Corporation which is hereby acknowledged [39]; Fig. 15b is courtesy of D. J. Donahue.

10 The critical, non-AMS issue relates to sample validity. The originally agreed upon sampling protocol was to have involved seven laboratories, two measurement techniques (decay and atom [AMS] counting), and multiple samples representing different regions of the cloth. Shortly before the event, however, the scheme was changed to restrict the number of laboratories (all AMS) and the number of samples to three, *all taken from the same location.* The sampling location, near a corner of the Shroud, and near an area damaged by the fire of 1532 AD, is considered an unfortunate choice, because of the possibility of exogenous carbon from the fire, repairs, and organic contamination from handling through the ages [40, 41]. Organic contamination cannot be dismissed. Recent observations indicate the presence of a bacterially-induced “bioplastic” coating on Shroud fibers, as has sometimes been found on mummy wrapping fabric (leading to erroneous dates). According to [42] (Gove, et al.), such bioplastic contamination would not have been removed by the conventional pre-treatment methods applied to the Shroud samples. Qualitatively, such contamination would lead to a more recent date; quantitatively, if the contamination were all from the 16th Century, it would need to represent roughly 70 % of the carbon present, to shift a first century date to the observed result. (For recent, late 20th Century contamination, roughly 40 % contamination carbon would be required.) In a 2002 review article posted to the shroud website, *www.shroud.com/pdfs/rogers2.pdf*, [38], Rogers and Arnoldi question the bioplastic hypothesis, on the basis of detailed chemical analysis of fibers from the “Raes sample” which was taken from a region adjacent to that of the ^14^C samples. Quantitatively, these authors suggest that the coating would contribute only a few percent to the sample carbon; qualitatively, they believe that it is a polysaccharide gum (probably Gum Arabic) that would be removed by the ^14^C pretreatment chemistry. Nevertheless, Rogers and Arnoldi question the validity of the ^14^C sample, partly because of the presence of cotton and other chemical differences between the adjacent (Raes) sample and the main shroud material.

11 There is a profound difference between background-limited decay counting and blank-limited AMS, that may not be widely appreciated. Although the ultimate limitation in each case is “B” variability, when B represents the instrumental background it tends to be reasonably well controlled, and under the best of circumstances, Poissonian [45]. An extra degree of caution is needed, however, when the limiting “B” is an isotopic-chemical blank. At best, it might be assumed normal; then replicate-based detection tests and confidence intervals can be constructed using Student’s-t. If the blank does not represent a homogeneous or stationary state (as a reagent blank, well-mixed environmental or biological compartment, etc.), such tests and intervals can be totally misleading. Non-stationary blanks may exhibit (geochemically meaningful) structure, or they may be erratic, reflecting a transient source of contamination [46].

12 Molecular dating” of the pure cellulose fraction of the Shroud, or of the associated pollen, could furnish an interesting consistency test for the published radiocarbon date. It would be especially interesting to put a “time stamp” on pollen whose point of origin has already been ascribed to a location 10 km to 20 km east and west of Jerusalem [47]. Such measurements are made feasible by the reduction of requisite sample sizes by a factor of ten or more, from what AMS ^14^C dating required sixteen years ago. The question of non-contemporaneous fiber from 16th Century repairs, for example, could be addressed by new ^14^C measurements on just 100 µg of fibers (≈50, 1 cm linen fibers) from the main part of the Shroud. The expected standard uncertainty would be equivalent to approximately 120 radiocarbon years ([43], Eq. 1).

13 The observed (Fig. 20) vs inferred (Fig. 19) paths of the smoke plume present an interesting contrast. The TOMS satellite image shows the smoke approaching the southern tip of Greenland on 3, 4 August 1994 and departing toward Iceland on 6 August. The back-trajectory model employed in Ref. [52] places the approach at a somewhat higher latitude, and of course provides no departure information.

14 The micro-molar ^14^C capability was essential for this work because of the extremely small concentrations of particulate carbon in the surface snow, especially during the winter (<10 µg C/kg snow).

15 Microanalytical methods, such as LAMMS, are crucial for gaining chemical insight on individual particles, or when only very small snow (or ice) samples of remote aerosols are available, or needed for high resolution studies. In contrast to the ng capability of the most sensitive bulk analysis techniques, LAMMS, can provide useful chemical data on as little as 20 pg of carbon species [57].

16 Although a “true” (Certified) EC value may be beyond reach, compatibility of results from laboratories using the same method suggests the possibility of method-specific (“operational”) EC *Reference Values* for this SRM.
